# Editorial: Best surgical treatment of breast cancer managed primarily with neoadjuvant medical therapy

**DOI:** 10.3389/fsurg.2025.1638260

**Published:** 2025-08-07

**Authors:** Ugo Marone

**Affiliations:** Breast Surgery Unit, IRCCS Fondazione “G. Pascale”, Naples, Italy

**Keywords:** neoadjuvant chemotherapy, breast surgery, conservative mastectomies, conservative surgery, axillary lymph node dissection (ALND)

**Editorial on the Research Topic**
Best surgical treatment of breast cancer managed primarily with neoadjuvant medical therapy

## The rationale for neoadjuvant chemotherapy

Neoadjuvant chemotherapy (NAC) has currently taken on a predominant role in the treatment of patients with breast cancer.

While in past years this type of treatment was mainly used to make locally advanced breast tumors operable, therefore for reduction of the tumor size, currently, it is the biological profile of the tumor that indicates the use of neoadjuvant therapy thanks to the increase in the constellation of drugs that can be used and the optimal pathological response, in many cases complete, that can be obtained in certain bioprofiles, such as triple negative and HER2 positive (1).

## Radiological consideration

For the breast surgeon, it is essential to verify two main points: verifying the extent of the residual tumor during post-treatment radiological diagnostic evaluation, comparing it with the initial state, and establishing the most appropriate surgical intervention in terms of conservative surgery vs. mastectomy procedures with simultaneous plastic reconstruction (2, 3).

In our research topic we have tried to collect scientific works specifically focused on the study of radiological methods that can provide a better definition of residual tumor disease after neoadjuvant therapy, such as ultrasound with elastosonography and nuclear magnetic resonance associated with predictive models also obtained with artificial intelligence procedures.

## Surgical approaches (conservation vs. mastectomy)

Regarding the surgical point of view, surgical treatment approach for breast cancer has changed from “maximum tolerable” to “minimum effective”. Evaluating the response of breast cancer patients to NAC before surgery is vital for adapting personalized surgical procedures and treatment approaches.

Crucial points of topic's papers were to establish selection criteria to designate patients for conservative or mastectomy procedures and understand if subcutaneous mastectomies are the most appropriate surgical procedures in those categories of patients compared to conservative surgery, and if so, in which instances.

Patients with isolated residual tumors by unifocal or initially multifocal tumors within the same quadrant, with concentric narrowing pattern of clinical-radiological response, are prone to conservative surgery.

In the instance of multicentric or advanced disease at the outset with patchy or multifocal regression, satellite lesions or uneven reduction of tumor volume, also in relation to the size and shape of the involved breast, are better handled surgically by conservative mastectomies, combined with immediate breast reconstruction, not done immediately only in cases of inflammatory breast cancer or when there is direct skin infiltration by the residual tumor. Several retrospective studies have demonstrated that all this doesn't increase the risk of local relapse or negatively affect long-term survival, while significantly improving the psychophysical recovery from the disease.

## Axillary management

The utilization of NAC has also altered the method to axillary lymph node management in breast cancer. Axillary lymph node dissection (ALND) is performed where strictly necessary, reducing the onset of the known possible complications associated with this procedure, emphasizing its prognostic role.

ALND can be omitted for patients whose positive nodes become negative and the appropriate treatment also for these patients is represented by the sentinel lymph node biopsy (SLNB), with the use of dual tracer sampling and removing a minimum of three lymph nodes, or performing SLNB with targeted axillary dissection, to lessen the false negative rate related to this procedure after NAC.

The accurate definition of the residual tumor volume at lymph node level, as a response to NAC, capable of influencing subsequent ALND, is still under study, bearing in mind that the macroscopic involvement of the lymph node detected by intraoperative molecular examination represents one of the most valid opportunities to more stringently select patients deserving of ALND (4).
